# Proteomic identification and functional characterization of MYH9, Hsc70, and DNAJA1 as novel substrates of HDAC6 deacetylase activity

**DOI:** 10.1007/s13238-014-0102-8

**Published:** 2014-10-15

**Authors:** Linlin Zhang, Shanshan Liu, Ningning Liu, Yong Zhang, Min Liu, Dengwen Li, Edward Seto, Tso-Pang Yao, Wenqing Shui, Jun Zhou

**Affiliations:** 1 State Key Laboratory of Medicinal Chemical Biology, College of Life Sciences, Nankai University, Tianjin, 300071 China; 2 High-throughput Molecular Drug Discovery Center, Tianjin Joint Academy of Biotechnology and Medicine, Tianjin, 300457 China; 3 Molecular Oncology Program, H. Lee Moffitt Cancer Center and Research Institute, Tampa, FL 33612 USA; 4 Department of Pharmacology and Cancer Biology, Duke University, Durham, NC 27710 USA

**Keywords:** HDAC6, substrate, lysine acetylation, quantitative proteomics, interaction

## Abstract

Histone deacetylase 6 (HDAC6), a predominantly cytoplasmic protein deacetylase, participates in a wide range of cellular processes through its deacetylase activity. However, the diverse functions of HDAC6 cannot be fully elucidated with its known substrates. In an attempt to explore the substrate diversity of HDAC6, we performed quantitative proteomic analyses to monitor changes in the abundance of protein lysine acetylation in response to HDAC6 deficiency. We identified 107 proteins with elevated acetylation in the liver of HDAC6 knockout mice. Three cytoplasmic proteins, including myosin heavy chain 9 (MYH9), heat shock cognate protein 70 (Hsc70), and dnaJ homolog subfamily A member 1 (DNAJA1), were verified to interact with HDAC6. The acetylation levels of these proteins were negatively regulated by HDAC6 both in the mouse liver and in cultured cells. Functional studies reveal that HDAC6-mediated deacetylation modulates the actin-binding ability of MYH9 and the interaction between Hsc70 and DNAJA1. These findings consolidate the notion that HDAC6 serves as a critical regulator of protein acetylation with the capability of coordinating various cellular functions.

**Electronic supplementary material:**

The online version of this article (doi:10.1007/s13238-014-0102-8) contains supplementary material, which is available to authorized users.

## **INTRODUCTION**

Reversible lysine acetylation, as an evolutionarily conserved protein posttranslational modification, regulates a variety of cellular processes including gene expression, enzyme activity, and protein-protein interactions. Although lysine acetylation was initially reported to regulate nuclear proteins such as histones and transcription factors (Allfrey et al., 1964; Gershey et al., 1968; Gu and Roeder, 1997), large-scale proteomic surveys have demonstrated that this modification is also prevalent outside the nucleus. The first global proteomic survey of reversible lysine acetylation described 37 acetylated proteins from the cytoplasmic fraction of HeLa cells and 133 from the mitochondria of the mouse liver (Kim et al., 2006). Subsequently, several groups reported a large number of acetylated non-nuclear proteins (Choudhary et al., 2009; Schwer et al., 2009; Zhao et al., 2010; Chen et al., 2012). Recently, two studies have extensively characterized the mitochondrial acetylome from the mouse liver via isotope labeling-based or label-free proteomic quantification (Hebert et al., 2013; Rardin et al., 2013).

In support of these proteomic surveys, several protein acetyltransferases and deacetylases, key regulatory enzymes of lysine acetylation, have been reported to localize in the cytoplasm or in the mitochondria (Peserico and Simone, 2011; Sadoul et al., 2011). In addition, many of these enzymes undergo nucleocytoplasmic transport (Peserico and Simone, 2011; Sadoul et al., 2011). Histone deacetylase 6 (HDAC6), a member of the class II HDAC family, is unique in being predominantly cytoplasmic (Verdel et al., 2000), which served as an early indication of the vital roles of HDAC6 in regulating lysine acetylation and relevant cellular pathways in the cytoplasm. Previous studies have documented several proteins as the substrates of HDAC6, including α-tubulin (Hubbert et al., 2002; Zhang et al., 2003), Hsp90 (Kovacs et al., 2005), cortactin (Zhang et al., 2007), peroxiredoxins (Parmigiani et al., 2008), β-catenin (Li et al., 2008), Ku70 (Subramanian et al., 2011), Tat (Huo et al., 2011), and survivin (Riolo et al., 2012).

Through the deacetylase activity, HDAC6 participates in a wide range of cellular processes, such as cell motility (Hubbert et al., 2002; Zhang et al., 2007; Li et al., 2011; Li et al., 2014), cell proliferation (Li et al., 2008), and cell survival (Subramanian et al., 2011; Riolo et al., 2012). The widespread roles of HDAC6 are manifested in impaired immune response, bone development, and glucocorticoid receptor translocation as well as abnormal emotional behaviors observed in HDAC6 knockout mice (Zhang et al., 2008; Fukada et al., 2012). However, these phenotypes cannot be fully elucidated with the limited number of the known HDAC6 substrates. Thus, it would be a plausible speculation that a set of uncharacterized HDAC6 substrates are present in the cytoplasm. A global and detailed analysis of the endogenous targets of HDAC6 will be undoubtedly helpful to decipher its diverse functions.

In the present study, we report the first quantitative proteomic analysis of lysine-acetylated proteins in the cytoplasmic fraction of the mouse liver in response to HDAC6 deficiency. A simplified proteomic workflow combining protein immunopurification, peptide isotope labeling, and 1D LC-MS/MS analysis identified 107 proteins that were hyperacetylated in HDAC6 knockout mice. The subsequent biochemical assays on selected substrates verified that HDAC6 interacts with and deacetylates MYH9, Hsc70, and DNAJA1. Moreover, our study implicates that HDAC6-mediated deacetylation plays pivotal roles in regulating the functions of the new substrates. Therefore, our findings expanded the knowledge on the HDAC6 substrate pool and its functional diversity, which might be a valuable resource for more in-depth dissection of HDAC6-mediated pathways in the cytoplasm.

## **RESULTS**

### **Strategy for lysine-acetylation proteomic analysis of wild-type and HDAC6 knockout mice**

**Figure 1:**
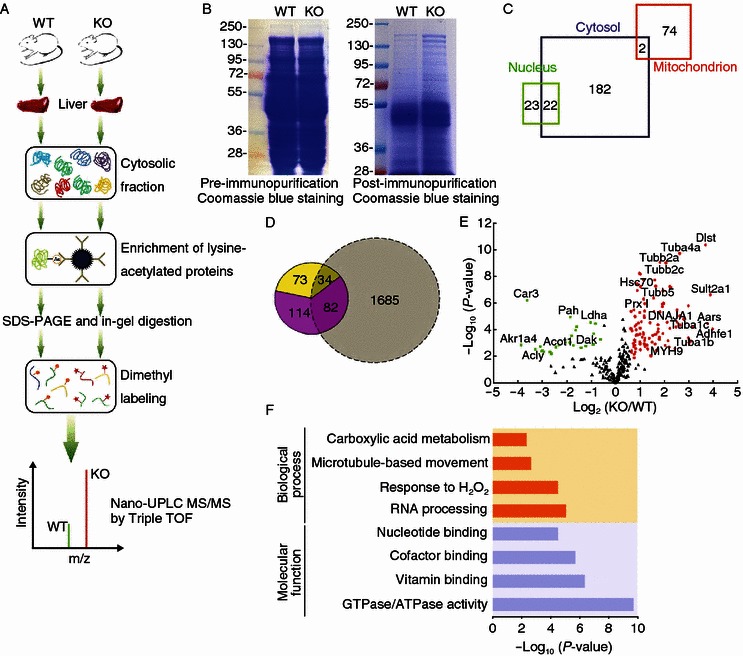
**Experimental strategy for the identification of HDAC6-regulated acetylated proteins in mouse liver tissues**. (A) Schematic of the experimental workflow. Cytoplasmic proteins extracted from the liver tissues of wild-type and HDAC6 knockout mice were subjected to immunopurification, SDS-PAGE, in-gel digestion, and isotope labeling followed by 1D LC-MS/MS analysis. (B) Coomassie blue-stained SDS-PAGE of cytoplasmic protein extracts prior to (left panel) or post (right panel) immunopurification with an antibody against acetylated lysine (AcK). (C) Cellular compartment distribution of 303 quantified proteins in our dataset. (D) Overlap of lysine-acetylated protein identification between our dataset (in color) and a large MEF acetylome dataset reported by the Zhao’s group (Chen et al., 2012) (grey). The fraction of potential HDAC6 substrates (yellow) is entailed in the total of quantified proteins in our study (pink). (E) Scatter plot showing the relative quantification of lysine-acetylated proteins in relation to significance of the protein ratio. Red and green dots represent proteins with increased or decreased acetylation abundance in HDAC6 knockout *vs*. wild-type mice, respectively. Black dots represent proteins with no significant changes in acetylation levels. (F) Representative biological processes and molecular functions significantly enriched in proteins with altered acetylation levels in HDAC6 knockout *vs*. wild-type mice, suggested by ontology annotation

To identify cytoplasmic proteins with acetylation levels changed in response to HDAC6 deficiency, we prepared cytoplasmic fractions from the liver tissues of wild-type and HDAC6 knockout mice (Fig. 1A). Liver was chosen because of the relatively high abundance and activity of HDAC6 observed in this tissue compared with others (Fig. S1A). The protein extracts were then subjected to immunoprecipitation with anti-acetyl-lysine antibodies. The enriched lysine-acetylated proteins from wild-type and HDAC6 knockout mice were subject to SDS-PAGE and concentrated into a single gel band prior to tryptic digestion (Fig. 1A). Relative quantification of proteins from different samples was achieved by dimethyl isotope labeling and MS according to previously published methods (Boersema et al., 2008; Boersema et al., 2009). Biological duplicates were prepared from two pairs of wild-type and HDAC6 knockout littermates, with forward or reverse isotope labeling. In each replicate, immunoprecipitation and protein digestion were performed in triplicate given that most experimental variation was introduced in these steps.

It is noteworthy that our workflow simplifies the proteomic analysis by targeting a limited number of lysine-acetylated proteins in the cytoplasm so that 1D LC-MS/MS is adequate for protein identification and quantification. It is recognized, however, that our workflow is unable to pinpoint the acetylation sites or directly measure changes of modified peptides in the acetylated proteins. Enzyme-mediated modification sites on numerous substrates could be revealed by large-scale proteomic analysis of enriched peptides. This peptide-centric strategy typically requires subcellular fractionation and 2D LC-MS/MS analysis, which were employed in the previous elegant studies of sirtuin 1 (SIRT1), SIRT3, and SIRT5 (Chen et al., 2012; Hebert et al., 2013; Park et al., 2013; Rardin et al., 2013). By adopting a less time-consuming proteomic workflow, we attempted to expedite our discovery phase and devote more effort to the downstream substrate validation and functional studies.

### **Plentiful cytoplasmic proteins are hyperacetylated in HDAC6 knockout mice**

Anti-acetyl-lysine immunoprecipitates prepared from the cytoplasmic extracts of the HDAC6 knockout mouse liver showed more abundant acetylation over a wide array of proteins than the wild-type tissue (Fig. 1B). To examine whether the above effect is due to HDAC6 deficiency-induced down-regulation of other deacetylase family members, such as HDAC3, HDAC4, HDAC5, HDAC7, HDAC9, HDAC11, and SIRT2, we performed immunoblotting to compare the expression of these deacetylases in wild-type and HDAC6 knockout mouse liver tissues. We found that HDAC6 deficiency did not significantly affect the expression of these deacetylases in the mouse liver (Fig. S1B). These data indicate that HDAC6 is an important regulator of lysine acetylation in the cytoplasm.

From the six replicates of proteomic analysis, 303 high-confidence proteins were quantified in at least four replicates. Their knockout/wild-type ratios indicate relative changes of protein acetylation levels in the livers of HDAC6 knockout mice *vs*. wild-type mice. Statistical tests were performed to identify significantly changed lysine-acetylated proteins in response to HDAC6 deficiency (*P* < 0.01, over 1.5-fold change, Datasheet S1). The initial proteomic identification and quantification reports for all six replicates were provided in Datasheet S2. Cellular compartment analysis of the confidently quantified proteins in our study suggested the enrichment of large portion of cytoplasmic (68%) and mitochondrial (25%) proteins with the anti-acetyl-lysine antibodies (Fig. 1C). As a result of nuclear depletion, the fraction of nuclear proteins in our dataset (15%) was significantly lower compared to a previous comprehensive acetylome analysis of SIRT1 knockout MEFs (28% nuclear proteins) (Chen et al., 2012). This previous analysis of SIRT1 knockout MEFs represents one of the highest numbers of acetylated proteins and acetylated sites reported in mammals. Although our dataset was relatively small and only included quantifiable proteins selected with stringent criteria, 187 proteins from our analysis did not overlap with the ~1800 acetylated proteins reported earlier (Fig. 1D).

In addition, we compared our protein identifications with two latest acetylome analyses of mitochondrial proteins from the liver of SIRT3 knockout mice (Hebert et al., 2013; Rardin et al., 2013) and also found unique proteins in our dataset (Fig. S2). Differences in sample source, protein extraction and fractionation, antibody enrichment strategy (protein *vs*. peptide), LC separation (1D *vs*. 2D), and MS platform could all result in differences in protein identification. Therefore, our study made a distinct contribution to the acetyl proteomic atlas of the mouse liver, and more importantly, uncovered a subset of acetylated proteins potentially regulated by HDAC6.

Out of the 303 high-confidence proteins, 107 proteins showed increased acetylation abundance in HDAC6 knockout mice and 27 proteins were decreased in acetylation levels (Fig. 1E). Known substrates of HDAC6, such as α-tubulin isoforms and peroxiredoxin, were found in the up-regulated subset with a fold-change of 1.6 up to 9.1 (*P* < 0.01). HDAC6 deficiency dramatically increased the acetylation level of a number of cytoplasmic and mitochondrial proteins which are regarded as the potential new substrates of this deacetylase (Fig. 1E; Datasheet S1). These proteins with molecular functions of nucleotide/cofactor/vitamin binding or GTPase/ATPase activity are significantly enriched in processes related to microtubule-based movement, carboxylic acid catabolism, and oxidative stress response as well as RNA processing (enrichment *P* < 0.01), suggesting the large substrate diversity of HDAC6 (Fig. 1F).

### **HDAC6 deacetylates selected substrate candidates**

As we were interested in the discovery of new cytoplasmic substrates, we excluded all known HDAC6 substrates such as tubulin and peroxiredoxin I (Prx I) and mitochondrial enzymes such as Dlst and Adhfe1 that were co-purified in the immunoprecipitation step. We selected three cytoplasmic proteins with 1.8 to 4.4-fold increase of the acetylation level in response to HDAC6 deficiency as potential new substrates of HDAC6, including MYH9, Hsc70, and DNAJA1, which have been previously reported to undergo reversible lysine acetylation (Choudhary et al., 2009).

**Figure 2:**
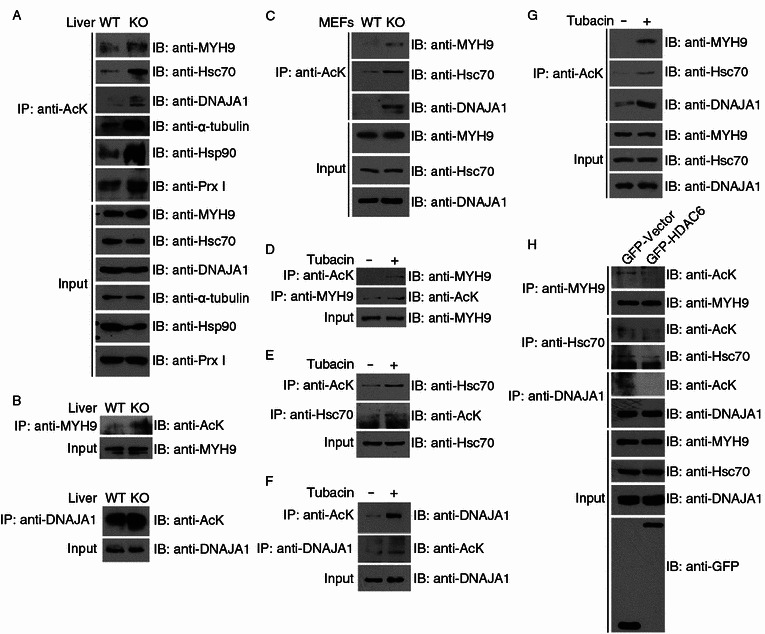
**HDAC6 deacetylates selected substrate candidates**. (A) Soluble cytoplasmic proteins were extracted from the liver tissues of wild-type and HDAC6 knockout mice, and immunoprecipitation was performed with anti-AcK antibodies. The immunoprecipitates and tissue extracts were then immunoblotted with the indicated antibodies. (B) Protein samples were prepared as in (A), and immunoprecipitation was performed with anti-MYH9 or anti-DNAJA1 antibodies. The immunoprecipitates and tissue extracts were then immunoblotted with the indicated antibodies. (C) Total cell lysates were prepared from wild-type and HDAC6 knockout MEFs. Anti-AcK immunoprecipitates and cell lysates were immunoblotted with antibodies against MYH9, Hsc70, or DNAJA1. (D–F) 293T cells were treated with DMSO (−) or tubacin (+) for 8 h. Anti-AcK, anti-MYH9, anti-Hsc70, or anti-DNAJA1 immunoprecipitates and total cell lysates were immunoblotted with the indicated antibodies. (G) Cytoplasmic proteins were extracted from 293T cells treated with DMSO (−) or tubacin (+) for 8 h. Anti-AcK immunoprecipitates and cytoplasmic extracts were immunoblotted with antibodies against MYH9, Hsc70, or DNAJA1. (H) 293T cells were transfected with GFP-HDAC6 or GFP alone. Immunoprecipitation and immunoblotting were then performed with the indicated antibodies

Multiple biochemical assays were employed for substrate validation. Immunoprecipitation was first performed on liver protein extracts of wild-type and HDAC6 knockout mice with anti-acetyl-lysine antibodies followed by immunoblot analysis of individual proteins (forward validation). Akin to several known HDAC6 substrates such as α-tubulin, Hsp90, and Prx I, MYH9, Hsc70, and DNAJA1 were immunoprecipitated by the anti-acetyl-lysine antibody in higher amount from HDAC6 knockout mice than wild-type mice (Fig. 2A), suggesting that their acetylation abundance was elevated upon HDAC6 deficiency. In the reciprocal experiment, liver extracts were immunoprecipitated with antibodies against individual putative substrates, followed by detection of their acetylation abundance (reverse validation). MYH9 and DNAJA1 from HDAC6 knockout mouse liver showed increased acetylation signal when compared with those from the wild-type tissue (Fig. 2B), whereas acetylated Hsc70 was undetectable in the reverse validation experiment.

Mouse embryonic fibroblasts (MEFs) generated from wild-type and HDAC6 knockout mice were used for further verification. By the forward validation method using total cell lysates, we observed elevated acetylation abundance of MYH9, Hsc70, and DNAJA1 from HDAC6 knockout MEFs (Fig. 2C).

To assess whether the deacetylation of the substrate candidates is dependent on HDAC6 activity, we treated 293T cells with the HDAC6-specific inhibitor tubacin (Haggarty et al., 2003) before analyzing the changes in substrate acetylation level. The efficiency of drug treatment was demonstrated by effective inhibition of α-tubulin deacetylation by HDAC6 (Fig. S3A). Results of forward and reverse validation showed that tubacin treatment resulted in elevated acetylation abundance of all three substrate candidates in 293T cells (Fig. 2D–F). Treatment of cells with trichostatin A (TSA), an inhibitor of the class I and II HDACs (Yoshida et al., 1995), also increased the acetylation levels of the putative substrates in a dose-dependent manner, whereas treatment with sodium butyrate (NaB), an HDAC inhibitor not affecting HDAC6 (Barlow et al., 2001), led to no change in the acetylation of MYH9 or Hsc70 (Fig. S3B and S3C). These data strongly suggest that HDAC6 is the major, if not the only, regulator of acetylation of these three substrate candidates.

Given that the proteomic identification of the three substrate candidates was from the cytoplasmic fractions of mouse liver tissues, we further performed the experimental validations with cytoplasmic fractions of 293T cells. As shown in Fig. 2G, all the three substrate candidates showed elevated acetylation in response to tubacin treatment. We also found that overexpression of GFP-HDAC6 significantly decreased the acetylation levels of MYH9, Hsc70, and DNAJA1 (Fig. 2H). Taken together, our multi-facet validation assays using the mouse liver, MEFs, and cell lines demonstrate that HDAC6 deacetylates the three new substrates.

### **HDAC6 interacts with the new substrates**

**Figure 3:**
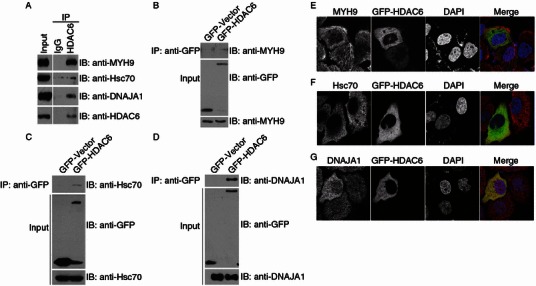
**HDAC6 interacts with the new substrates**. (A) 293T cell lysates were incubated with control IgG or anti-HDAC6 antibodies. The immunoprecipitates and cell lysates were then immunoblotted with the indicated antibodies. (B–D) 293T cells were transfected with GFP-HDAC6 or GFP alone. Anti-GFP immunoprecipitates and cell lysates were immunoblotted with the indicated antibodies. (E–G) Immunofluorescence confocal images of HeLa cells transfected with GFP-HDAC6 and stained with anti-MYH9 (E), anti-Hsc70 (F), or anti-DNAJA1 (G) antibodies and the DNA dye DAPI

Given that physical interactions are required for enzyme-substrate pairs as reported for HDAC6 and its known substrates (Hubbert et al., 2002; Zhang et al., 2003; Kovacs et al., 2005; Zhang et al., 2007), we examined by immunoprecipitation and immunofluorescence microscopy whether HDAC6 interacts with the new substrates. As shown in Fig. 3A, endogenous MYH9, Hsc70, and DNAJA1 were present in the immunoprecipitates of endogenous HDAC6 in 293T cells.

To further characterize the interaction of HDAC6 with the novel substrates, a series of transfection and immunoprecipitation were performed. The anti-GFP antibodies specifically coprecipitated MYH9 (Fig. 3B), Hsc70 (Fig. 3C), and DNAJA1 (Fig. 3D) from 293T cells transfected with GFP-HDAC6 but not GFP alone. Collectively, these data suggest that both endogenous and exogenously expressed HDAC6 interact with the new substrates.

Next, we examined the subcellular localization of HDAC6 and the new substrates by immunofluorescence confocal microscopy. A fraction of MYH9 diffusely present in the cytoplasm of HeLa cells showed significant colocalization with HDAC6 (Fig. 3E), yet another fraction, which distributed to the actin filaments, barely colocalized with HDAC6 (Fig. 3E). Hsc70 resided predominantly in the cytoplasm and showed apparent colocalization with HDAC6 (Fig. 3F). DNAJA1 diffusely distributed both in the nucleus and in the cytoplasm and partially colocalized with HDAC6 in the cytoplasm (Fig. 3G). Together, these results demonstrate that HDAC6 interacts with the three new substrates and colocalizes with each substrate to a certain level.

### **HDAC6-mediated deacetylation of MYH9 downregulates its actin-binding activity**

**Figure 4:**
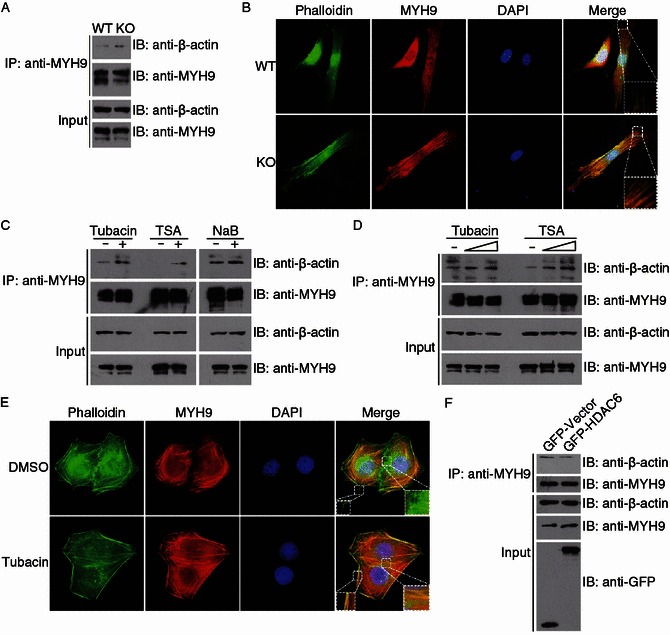
**HDAC6-mediated deacetylation of MYH9 downregulates its actin-binding activity**. (A) Lysates from wild-type and HDAC6 knockout MEFs were immunoprecipitated with anti-MYH9 antibodies. The immunoprecipitates and cell lysates were subjected to immunoblotting with anti-β-actin or anti-MYH9 antibodies. (B) Immunofluorescence images of wild-type and HDAC6 knockout MEFs stained with the anti-MYH9 antibody, phalloidin, and DAPI. (C) 293T cells were treated with tubacin, TSA, or NaB for 8 h. Anti-MYH9 immunoprecipitates and cell lysates were immunoblotted with anti-β-actin or anti-MYH9 antibodies. (D) 293T cells were treated with tubacin or TSA (1 or 5 μmol/L) and examined as in (C). (E) Immunofluorescence images of HeLa cells treated with DMSO or tubacin and stained with the anti-MYH9 antibody, phalloidin, and DAPI. (F) 293T cells were transfected with GFP-HDAC6 or GFP alone. Anti-MYH9 immunoprecipitates and cell lysates were immunoblotted with the indicated antibodies

MYH9 is known to associate with actin filaments through its globular head domain (Sutoh, 1982). We thus investigated whether the deacetylation of MYH9 by HDAC6 affects its actin-binding ability. Immunoprecipitation assay revealed that MYH9 interacted more strongly with actin in HDAC6 knockout MEFs as compared to wild-type MEFs (Fig. 4A). In addition, HDAC6 knockout MEFs exhibited stronger colocalization between MYH9 and actin, especially near the cell periphery (Fig. 4B). By immunoprecipitation assays, we further found that the actin-binding ability of MYH9 was increased by treatment with tubacin and TSA, but not NaB (Fig. 4C and 4D). Tubacin also led to significantly enhanced colocalization of MYH9 and actin, particularly near the cell periphery and the cell-cell junctions (Fig. 4E). Furthermore, overexpression of GFP-HDAC6 markedly attenuated the actin-binding affinity of MYH9 (Fig. 4F). Collectively, these results demonstrate that HDAC6-mediated deacetylation of MYH9 downregulates its actin-binding activity.

### **HDAC6-mediated deacetylation of Hsc70 and DNAJA1 are critical for their interaction**

**Figure 5:**
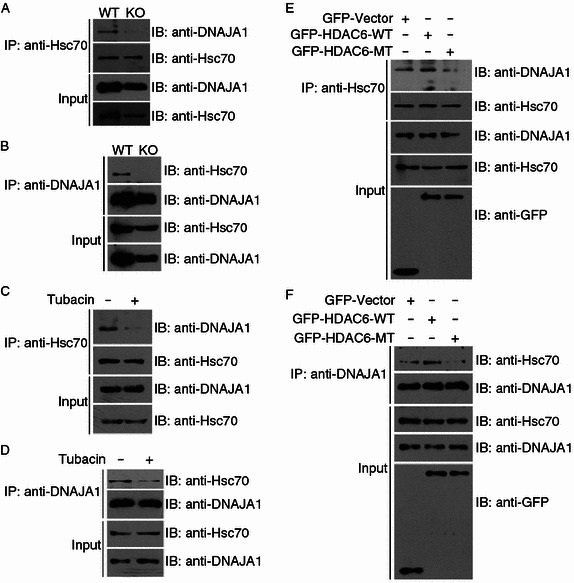
**HDAC6-mediated deacetylation of Hsc70 and DNAJA1 are critical for their interaction**. (A and B) Lysates from wild-type and HDAC6 knockout MEFs were immunoprecipitated with anti-Hsc70 (A) or anti-DNAJA1 (B) antibodies. The immunoprecipitates and cell lysates were then subjected to immunoblotting with anti-DNAJA1 or anti-Hsc70 antibodies. (C and D) 293T cells were treated with DMSO (−) or tubacin (+) for 8 h, and the lysates were immunoprecipitated with anti-Hsc70 (C) or anti-DNAJA1 (D) antibodies. The immunoprecipitates and cell lysates were then immunoblotted with anti-DNAJA1 or anti-Hsc70 antibodies. (E and F) 293T cells were transfected with GFP-HDAC6 wild-type (WT), the catalytically inactive mutant (MT), or GFP alone, and the lysates were immunoprecipitated with anti-Hsc70 (E) or anti-DNAJA1 (F) antibodies. The immunoprecipitates and cell lysates were then immunoblotted with anti-DNAJA1, anti-Hsc70, or anti-GFP antibodies

Hsc70 and DNAJA1 are known to function together to promote proper folding of proteins (Meacham et al., 1999). Our findings that HDAC6 deacetylates both Hsc70 and DNAJA1 prompted us to determine whether the association between these two proteins is influenced by HDAC6. By immunoprecipitation assays, we found that the interaction between Hsc70 and DNAJA1 was significantly decreased in HDAC6 knockout MEFs as compared to wild-type MEFs (Fig. 5A and 5B). In addition, treatment of cells with the HDAC6 inhibitor tubacin remarkably inhibited the interaction between these two proteins (Fig. 5C and 5D). Furthermore, overexpression of wild-type HDAC6, but not the catalytically inactive mutant that harbors H216A and H611A mutations (Grozinger et al., 1999), enhanced the interaction between Hsc70 and DNAJA1 (Fig. 5E and 5F). These data suggest that HDAC6-mediated deacetylation of Hsc70 and DNAJA1 are critical for their interaction.

## **DISCUSSION**

Detection of cytoplasmic HDAC6 substrates is often hindered by the high abundance of heavily acetylated histones and other proteins in the nucleus or mitochondria, and possibly low abundance of certain acetylated proteins in the cytoplasm. In this study, we attempted to circumvent these technical issues by combining subcellular fractionation, affinity purification, and high-resolution and high-accuracy mass spectrometry to specifically enrich and quantify acetylated proteins from the cytoplasmic fraction of the mouse liver.

Prior to our study, a handful of proteins were well characterized to be HDAC6 substrates, such as α-tubulin (Hubbert et al., 2002; Zhang et al., 2003), Hsp90 (Kovacs et al., 2005), and peroxiredoxins (Parmigiani et al., 2008). Our quantitative proteomic analysis confirmed the hyperacetylation of these known substrates in HDAC6 knockout mice and further uncovered more proteins with increased acetylation in response to HDAC6 deficiency. Notably, a few other known substrates including cortactin (Zhang et al., 2007), survivin (Riolo et al., 2012), β-catenin (Li et al., 2008), and Ku70 (Subramanian et al., 2011) were not detected in our study, possibly due to the low efficiency of the anti-acetyl-lysine antibodies in enriching these particular proteins. In addition, the predominant nuclear localization of survivin in response to HDAC6 deficiency (Riolo et al., 2012), the dependence on epidermal growth factor-stimulation of β-catenin deacetylation by HDAC6 (Li et al., 2008), and the variable expression levels of Ku70 in different cells and tissues, could collectively contribute to the difficulties in identifying these known substrates. An alternative workflow involving the enrichment of acetylated peptides coupled with extensive 2D-LC separation might render a more in-depth characterization of HDAC6-mediated acetylome dynamics.

**Figure 6:**
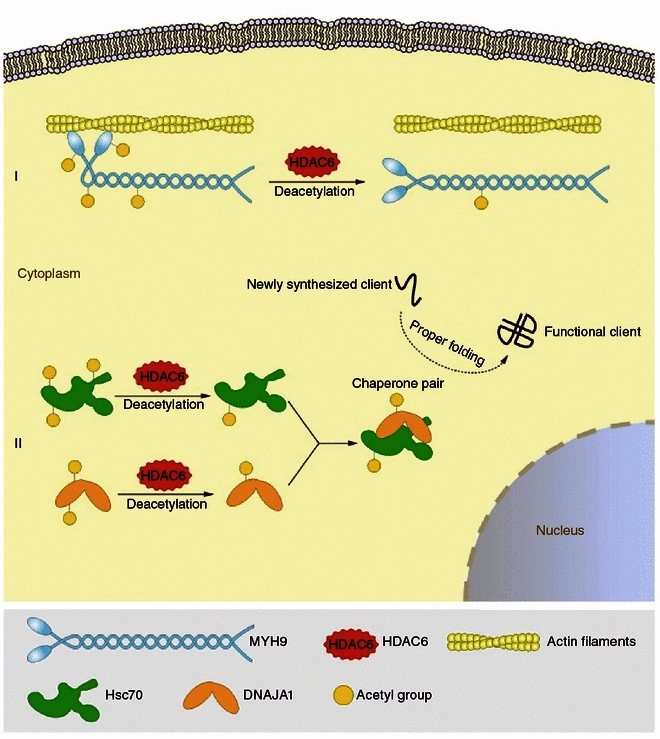
**Proposed model for HDAC6-mediated deacetylation and regulation of new substrates**. (I) HDAC6 deacetylates MYH9 and inhibits its actin-binding activity. (II) HDAC6 deacetylates Hsc70 and DNAJA1 and enhances their interaction, which might further modulate their function as a chaperone pair

HDAC6-mediated deacetylation of α-tubulin has been reported to regulate microtubule-dependent cell motility (Hubbert et al., 2002; Tran et al., 2007). In addition, actin-based cell movement has been demonstrated to be modulated by HDAC6 via deacetylation of the actin-binding protein cortactin (Zhang et al., 2007). In this report, we demonstrate that HDAC6 deacetylates MYH9, another actin-binding protein that is part of non-muscle myosin IIA (NM IIA), and thereby negatively regulates the actin-binding affinity of MYH9 in cells (Fig. 6). Through the regulation of the actin cytoskeleton, NM IIA is known to modulate various cellular processes such as cell migration, cell adhesion, and cell polarity, in ATPase activity-dependent or -independent manners (Vicente-Manzanares et al., 2009). The ATPase activity of NM IIA is activated when MYH9 binds to actin through the globular head domain, and is further regulated by reversible phosphorylation of the regulatory light chain (Somlyo and Somlyo, 2003). However, this reversible phosphorylation has been shown to have little or no effect on the actin-binding affinity of MYH9 (Sellers et al., 1982). Interestingly, our findings indicate that the actin-binding activity of MYH9 is altered in response to its acetylation status, which may further influence the ATPase activity of NM IIA, at least at the initial activation stage. NM IIA has been shown previously to be essential for the formation and stability of the cell-cell junctions (Vicente-Manzanares et al., 2009). Intriguingly, our data show that inhibition of HDAC6 activity induces intensive distribution of MYH9 to the cell-cell junctions. This might provide an additional explanation for the inhibitory effects of HDAC6 inhibitors on cell movement.

It has been shown previously that Hsp90 hyperacetylation, resulting from HDAC6 inhibition, leads to the dissociation of Hsp90 from its co-chaperone p23 and the loss of the chaperone activity of Hsp90 (Kovacs et al., 2005). Likewise, in this study we show that HDAC6 deacetylates Hsc70, another chaperone protein, and its co-chaperone DNAJA1, and influences the interaction between Hsc70 and DNAJA1 (Fig. 6). Due to the lack of knowledge about the sites of Hsc70 and DNAJA1 modulated by HDAC6, although we speculate that the effect of HDAC6 on the Hsc70-DNAJA1 interaction might be attributed to HDAC6-mediated deacetylation, the structural change of HDAC6 might also play a role in regulating their interaction. It has been well defined that the binding of the co-chaperone to Hsc70 stimulates its ATPase activity (Bukau and Horwich, 1998), it is thus conceivable that HDAC6 plays a crucial role in modulating the functions of the DNAJA1/Hsc70 chaperone pair. Our results, together with previous findings, therefore strongly suggest that HDAC6 is a critical regulator of the chaperone network.

In conclusion, our study has identified three new substrates of HDAC6 in the cytoplasm and provided a resource of putative cytoplasmic targets of this deacetylase for further investigation. The wide substrate diversity of HDAC6 suggests that it may play profound roles in multiple interrelated processes and pathways in the cytoplasm. Considering that small molecule compounds targeting HDACs are currently undergoing clinical trials for the treatment of cancer and metabolic diseases, a better understanding of HDAC6-dependent cellular processes would be highly valuable for the evaluation of the clinical relevance and therapeutic efficacy of HDAC6 inhibitors.

## **MATERIALS AND METHODS**

### **Plasmids, chemicals, and antibodies**

The GFP-HDAC6 expression plasmid was constructed by cloning human HDAC6 cDNA into the pEGFPN1 vector, and the catalytically inactive mutant was generated by site-directed mutagenesis as described (Zhou et al., 2009; Li et al., 2014). Triethylammonium bicarbonate, formaldehyde-H_2_ (28% in H_2_O), sodium cyanoborohydride, ammonium hydroxide, trifluoroacetic acid, 4’,6-diamidino-2-phenylindole (DAPI), fluorescein-phalloidin, trichostatin A (TSA), and sodium butyrate (NaB) were purchased from Sigma-Aldrich. Formaldehyde-D_2_ (28% in D_2_O, Cambridge Isotope Laboratory), formic acid and acetonitrile (Merck), and C_18_ desalting microcolumns (Nest Group) were from the indicated sources. Tubacin was obtained from Stuart Schreiber (Harvard Medical School). Antibodies against α-tubulin, acetylated α-tubulin, and β-actin (Sigma-Aldrich), HDAC6, HDAC7, HDAC9, Hsp90, Prx I, MYH9, Hsc70, and DNAJA1 (Abcam), HDAC3, HDAC4, HDAC5, HDAC11, and SIRT2 (Santa Cruz Biotechnology), acetylated lysine (Cell Signaling Technology), and GFP (Roche), horseradish peroxidase-conjugated secondary antibodies (Amersham Biosciences), and rhodamine-conjugated secondary antibodies (Jackson ImmunoResearch Laboratories) were obtained from the indicated sources.

### **Mice and cells**

HDAC6 knockout mice (in 129/C57BL6 mixed genetic background) were generated and genotyped as described previously (Gao et al., 2007). HDAC6 heterozygous mice were intercrossed to generate wild-type and HDAC6 knockout littermates. Mouse embryonic fibroblasts (MEFs) were isolated and cultured as previously described (Gao et al., 2007). HeLa and 293T cells were cultured in DMEM medium supplemented with 10% fetal bovine serum at 37°C in a humidified atmosphere with 5% CO_2_. All mouse experiments were performed in accordance with the relevant institutional and national guidelines and regulations; the experiments were approved by the Animal Care and Use Committee of Nankai University, and all experiments conform to the relevant regulatory standards.

### **Cytoplasmic protein extraction from the mouse liver**

Mouse liver tissues were minced into small pieces with a razor blade and scissors, and transferred to a glass Dounce homogenizer. The minced tissues were suspended in ice-cold PBS (pH 7.5) supplemented with the protease inhibitor cocktail (Roche) and homogenized with 40 strokes on ice. The homogenized mixture was centrifuged at 1000 ×*g* at 4°C for 10 min. The supernatant was transferred to another centrifuge tube and subjected to further centrifugation at 10,000 ×*g* at 4°C for 30 min. The supernatant was collected as the cytoplasmic fraction and used for subsequent analysis. Biological replicates were obtained from two pairs of wild-type and HDAC6 knockout littermates.

### **Affinity enrichment and in-gel tryptic digestion of lysine-acetylated proteins**

For affinity enrichment of lysine-acetylated proteins, the cytoplasmic fractions isolated from mouse liver tissues were incubated with anti-acetyl-lysine agarose beads at 4°C for 12 h with gentle shaking. The supernatant was removed and the beads were washed five times with ice-cold PBS (pH 7.5). The bound proteins were eluted from the beads with 2% SDS in 50 mmol/L Tris-HCl buffer (pH 8.0) by boiling for 10 min. The eluted fractions were dried in a vacuum evaporator. The resulting proteins from the affinity enrichment were loaded onto SDS-PAGE and concentrated at the boundary between the stacking gel and the separation gel. The single protein band of each sample stained by Coomassie blue was excised from the gel, and subjected to standard in-gel tryptic digestion as previously described (Shevchenko et al., 2006). The extracted peptide solutions were evaporated in a vacuum evaporator prior to stable isotope labeling. For each biological replicate of protein extracts from wild-type and HDAC6 knockout mouse livers, affinity enrichment and protein digestion were performed in triplicate.

### **Stable isotope dimethyl labeling**

Tryptic peptides from each sample were redissolved in 100 mmol/L triethylammonium bicarbonate (pH 8.5). Subsequently, for light labeling, 4 µL of formaldehyde-H_2_ (4% in water) was added to the solution (1:25 *v/v*) and vortexed for 1 min, followed by the addition of freshly prepared sodium cyanoborohydride to reach a final concentration of 25 mmol/L. The resultant mixture was incubated with shaking for 1 h at room temperature. A total of 16 µL of ammonia (1%) was added to consume the excess formaldehyde. Finally, 8 µL of formic acid (5%) was added to acidify the solution. For heavy labeling, 4 µL of formaldehyde-D_2_ (4% in D_2_O) was added. The light and heavy dimethyl-labeled peptides derived from equal amount of wild-type and HDAC6 knockout mouse liver protein extracts were pooled and desalted using C_18_ microcolumns. Forward and reverse labeling was performed separately for independent biological replicates.

### **Nano-HPLC-MS/MS analysis**

The mixed isotope-labeled peptides were analyzed by nanoflow reverse-phase UPLC-ESI-MS/MS using an Eksigent Ultra Plus nano-HPLC (AB SCIEX) connected to a quadrupole time-of-flight (QqTOF) TripleTOF 5600 mass spectrometer (AB SCIEX). After injection, peptide mixtures were transferred onto the analytical column (C_18_ Acclaim PepMap100, 75 µm I.D. × 15 cm, 3 µm particle size, Dionex) and eluted with a gradient of 2% to 35% B (A: 2% acetonitrile/98% water/0.1% formic acid; B: 98% acetonitrile/2% water/0.1% formic acid) over 1 h. Mass spectra and tandem mass spectra were recorded in positive-ion and “high-sensitivity” mode (resolution ~35,000). The mass window for precursor ion selection was set to ± 1 m/z. Advanced information dependent acquisition (IDA) was employed for MS/MS collection of the 20 most abundant parent ions. The exclusion mass width was 50 mDa and the exclusion duration was 20 s.

### **Proteomic data analysis**

Data were processed with Protein Pilot Software v.4.0 (AB SCIEX) utilizing the Paragon and Progroup Algorithm (Shilov et al., 2007). The software performs automatic recalibration such that typical mass errors for MS and MS/MS data were below 10 ppm. The database employed was an IPI mouse protein database (version 3.87) supplemented with the trypsin sequence and common protein contaminant sequences. In the software algorithm, all modifications listed in UniMod are searched simultaneously with the tolerances specified as ±0.05 Da for peptides and MS/MS fragments (Shilov et al., 2007). Protein Pilot automatically clusters the identified proteins into protein groups sharing common peptides. Only protein identifications with >99% confidence were retained, resulting in FDR <1% as calculated by decoy database search. For quantification analysis, peptide dimethylation at amino-termini or lysine residues was specified to be the quantification method. Only peptides of unique sequences (not shared with other proteins) and free of miscleavages or variable modifications contributed to protein ratio calculation. Protein ratios and *P*-values based on at least two unique peptide ratios were calculated by the software. In each replicate, only proteins identified with above 99% confidence and quantified with at least two unique peptides are considered quantifiable proteins. As we prepared two biological replicates with each processed in triplicate for immunoprecipitation and LC-MS/MS analysis, proteins that are quantifiable in at least two out of the three experiments of each biological replicate were retained for comparative analysis. The ultimate average ratios of individual proteins in the liver extracts of wild-type mice *vs*. HDAC6 knockout mice (WT/KO) were derived from four to six independent experiments, and *P*-values indicating significance of their differences from unity were calculated using two-tail student’s *t*-tests.

### **Cell transfection and treatment**

Cells were transfected with plasmids by using the polyethyleneimine reagent (Sigma-Aldrich). After 24 h of transfection, cells were subjected to immunoprecipitation or immunofluorescence microscopy. HDAC inhibitors were used at the following concentrations for 8 h unless indicated otherwise: tubacin (5 μmol/L), TSA (5 μmol/L), and NaB (10 mmol/L).

### **Immunoblotting and immunoprecipitation**

Proteins were resolved by SDS-PAGE and transferred onto polyvinylidene difluoride membranes (Millipore). The membranes were blocked with 5% fat-free milk in Tris-buffered saline containing 0.1% Tween 20 and probed with primary antibodies and then horseradish peroxidase-conjugated secondary antibodies. The target proteins were visualized with enhanced chemiluminescence detection reagent following the manufacturer’s instructions (Pierce Biotechnology). For immunoprecipitation, protein samples were incubated with antibody-conjugated agarose beads overnight at 4°C. The beads were washed extensively and subjected to immunoblotting.

### **Immunofluorescence microscopy**

Cells grown on glass coverslips were fixed with 4% paraformaldehyde for 30 min at room temperature. Cells were then treated with 0.5% Triton X-100 in PBS for 20 min for permeabilization and blocked with 2% bovine serum albumin in PBS. Cells were then incubated in succession with the primary antibody and rhodamine-conjugated secondary antibody followed by staining with DAPI for 3 min. For some experiments, cells were also stained with fluorescein-phalloidin for the visualization of actin filaments. Coverslips were mounted with 90% glycerol in PBS, and images were obtained using an Axio Observer A1 fluorescence microscope (Carl Zeiss Inc) or a TCS SP5 confocal microscope (Leica).

## Electronic supplementary material

Below is the link to the electronic supplementary material.

Supplementary material 1 (PDF 248 kb)

Supplementary material 2 (XLS 847 kb)

Supplementary material 3 (XLS 588 kb)

## **Abbreviations**

DAPI: 4’,6-diamidino-2-phenylindole
DNAJA1: dnaJ homolog subfamily A member 1
HDAC6: histone deacetylase 6
Hsc70: heat shock cognate protein 70
MEFs: mouse embryonic fibroblasts
MYH9: myosin heavy chain 9
NaB: sodium butyrate
NM IIA: non-muscle myosin IIA
Prx: peroxiredoxin
TSA: trichostatin A
