# Supplementary material for: Proteomic identification and functional characterization of MYH9, Hsc70, and DNAJA1 as novel substrates of HDAC6 deacetylase activity
Source: Protein Cell. 2014 Oct 15;6(1):42–54. doi: 10.1007/s13238-014-0102-8 (PMC4286133; doi:10.1007/s13238-014-0102-8)
Supplement: Supplementary file 1 — Supplementary material 1 (PDF 248 kb) [file 13238_2014_102_MOESM1_ESM.pdf]

Supplementary Materials

Legends of Supplementary Figures and Datasheets:

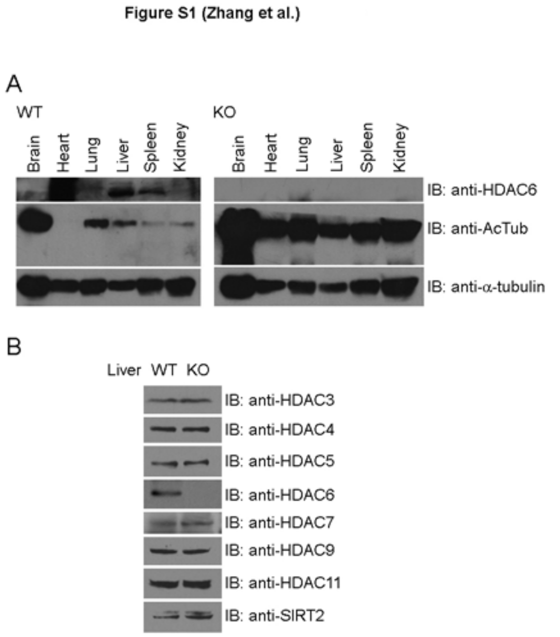

Figure S1. Immunoblot analysis of the expression of HDAC6 and other deacetylase family members in wild-type and HDAC6 knockout mouse tissues. (A) Proteins extracted from different tissues of wild-type or HDAC6 knockout mice were immunoblotted with antibodies against HDAC6, acetylated α-tubulin (AcTub), and α-tubulin, respectively. (B) Proteins extracted from liver tissues of wild-type or HDAC6 knockout mice were subjected to immunoblotting with the indicated antibodies.

Figure S2 (Zhang et al.)

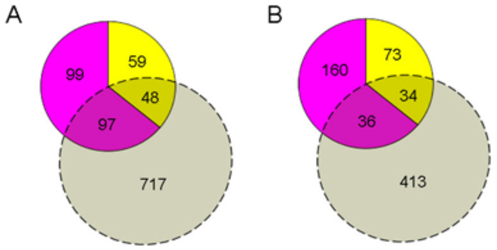

Figure S2. Overlap of lysine-acetylated protein identifications between our study and previous large-scale acetylome analyses. Overlap of lysine-acetylated protein identification between our dataset (in color) and mouse liver mitochondrial acetylome datasets reported by the Denu's group (Hebert et al., 2013) (grey) (A), and the Gibson's group (Rardin et al., 2013) (grey) (B). The fraction of potential HDAC6 substrates (yellow) is entailed in the total of quantified proteins in our study (pink).

Figure S3 (Zhang et al.)

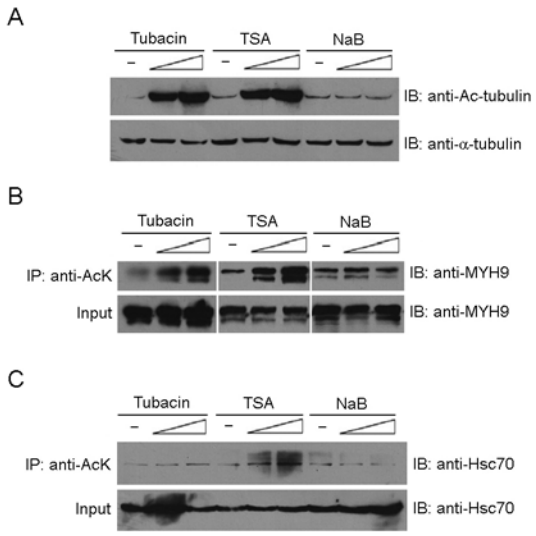

Figure S3. Effects of HDAC inhibitors on the acetylation levels of α-tubulin, MYH9, and Hsc70. 293T cells were treated with DMSO (-), tubacin (1 or 5 μM), TSA (1 or 5 μM), or NaB (1 or 10 mM) for 8 h. In (A), cell lysates were subjected to immunoblot analysis with antibodies against acetylated α-tubulin or α-tubulin. In (B) and (C), anti-AcK immunoprecipitates and total cell lysates were immunoblotted with antibodies against MYH9 (B) or Hsc70 (C).

Datasheet S1. Summary of the immunoprecipitated proteins quantified in at least four replicates.

Datasheet S2. Protein identification and quantification reports from six experimental replicates.

Supplementary References

Hebert, AS, Dittenhafer-Reed KE, Yu W, Bailey DJ, Selen ES, Boersma MD, Carson JJ, Tonelli M, Balloon AJ, Higbee AJ, et al. (2013) Calorie restriction and SIRT3 trigger global reprogramming of the mitochondrial protein acetylome. Mol Cell 49:186-199

Rardin MJ, Newman JC, Held JM, Cusack MP, Sorensen DJ, Li B, Schilling B, Mooney SD, Kahn CR, Verdin E, et al. (2013) Label-free quantitative proteomics of the lysine acetylome in mitochondria identifies substrates of SIRT3 in metabolic pathways. Proc Natl Acad Sci U S A 110:6601-6606
